# Perinatal Pandemic (H1N1) 2009 Infection, Thailand

**DOI:** 10.3201/eid1602.091733

**Published:** 2010-02

**Authors:** Wut Dulyachai, Jarika Makkoch, Pornpimol Rianthavorn, Mutita Changpinyo, Slinporn Prayangprecha, Sunchai Payungporn, Rachod Tantilertcharoen, Pravina Kitikoon, Yong Poovorawan

**Affiliations:** Ratchaburi Hospital, Ratchaburi, Thailand (W. Dulyachai, M. Changpinyo); Chulalongkorn University, Bangkok, Thailand (J. Makkoch, P. Rianthavorn, S. Prayangprecha, S. Payungporn, R. Tantilertcharoen**,** P. Kitikoon, Y. Poovorawan); King Chulalongkorn Memorial Hospital, Bangkok (P. Rianthavorn)

**Keywords:** Influenza, pandemic, perinatal, H1N1, viruses, Thailand, expedited, letter

**To the Editor**: Infection with influenza A pandemic (H1N1) 2009 has been reported worldwide following initial identification of the virus in April 2009 (*1*). The groups at highest risk for infection or influenza-related complications include pregnant women and children (*2*). We report a case of pandemic (H1N1) 2009 infection in a newborn whose mother became ill with pandemic (H1N1) 2009 during the perinatal period.

A newborn girl showed signs of respiratory distress. The relevant perinatal history was maternal illness with pandemic (H1N1) 2009 7 days before delivery. The infant, who had a birth weight of 1,560 grams, was delivered by emergency cesarean section after the mother experienced cardiopulmonary failure at the gestational age of 31 weeks. Apgar scores were 9 and 9 at 1 and 5 minutes, respectively. Physical examination at birth showed a premature infant girl with mild subcostal retraction. Oxygen saturation at room air was 91%–99%. Other results of the physical examination were unremarkable.

Initial management included routine care for premature infants. On the basis of the perinatal history, a throat swab specimen was collected for pandemic (H1N1) 2009 testing by PCR and oseltamivir, 6 mg, was administered every 12 hours (4 mg/kg/day). The specimen obtained from the throat swab was positive for pandemic (H1N1) 2009 by real-time PCR. The infant required oxygen supplementation. At day 2 of life, acute renal failure with an elevated plasma creatinine level of 1.1 mg/dL developed in the infant. Chest radiograph showed minimal pulmonary infiltrations. She was started on cefotaxime for suspected sepsis. Oseltamivir dosage was adjusted based on the glomerular filtration rate estimated by the formula of Schwartz et al. (*3*) of 10.5 mL/min/1.73m^2^ to 3 mg every 12 hours to complete 10 doses (2 mg/kg/day).

Infection of the patient was confirmed by real-time reverse transcription–PCR of the throat swab specimen and by a 4× increase in antibodies against the virus by hemagglutination inhibition test (HI). Antibody titers against pandemic influenza (H1N1) 2009 by HI with turkey erythrocytes (*4*) on days 10, 24, and 42 of life were 10, 160, and 320, respectively (Figure). At day 4 of life, repeated PCR performed on a throat swab specimen was negative for pandemic (H1N1) 2009. Oxygen supplementation was gradually decreased and finally discontinued. Her room air oxygen saturation was 95%–98%. Her clinical symptoms gradually improved. Hemoculture was negative after 72 hours. The antimicrobial drugs were given over an 8-day course. Plasma creatinine decreased to 0.9 mg/dL and 0.6 mg/dL at days 6 and 7 of life, respectively. Her average urine output was 2–3 mL/kg/h. She was discharged at the age of 28 days with a body weight of 2,070 grams.

**Figure:**
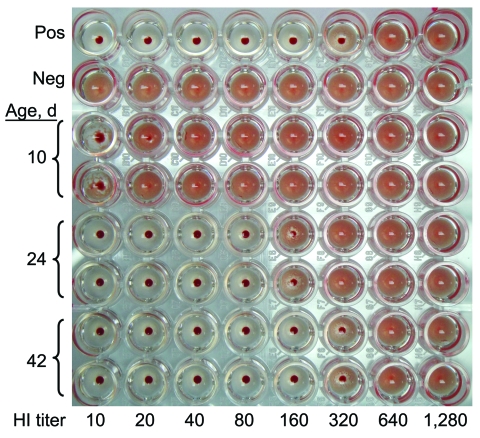
Antibody titer against influenza A pandemic (H1N1) 2009 by hemagglutination inhibition (HI) test on days 10, 24, and 42 of life of the patient.

Pregnant women are one of the highest risk groups for influenza A infection and influenza-associated complications, including increased maternal and perinatal illness and death rates (*5*). Thus, pregnant women are given first priority to receive influenza vaccination. When influenza develops in mothers during the perinatal period, newborns can be infected transplacentally during maternal viremia or by respiratory droplets after birth. Transplacental infection of influenza A is rare (*5*), however, and there have been only a few case reports (*6**,**7*). Viremia is more frequent and more extensive in pregnant women due to depressed cell-mediated immune response during the pregnancy (*8*). Our patient was likely infected in utero because she was delivered by cesarean section and was never exposed to her mother, who required intensive cardiopulmonary support at the time of delivery. (The mother died from respiratory failure 7 days after the cesarean section.)

Clinical manifestations in this patient, including respiratory distress and acute renal failure, were nonspecific. The high plasma creatinine level in the newborn sometimes reflects the mother’s plasma creatinine level (*9*). However, kidney function of the mother of the newborn was within normal limits at the time of cesarean section; plasma creatinine level of 0.7 mg/dL. An elevated plasma creatinine level is observed frequently in premature infants due to immaturity of the kidney tissue and will usually decrease within a few weeks. Oseltamivir was administered with dose adjustment based on the infant’s estimated g**lomerular filtration rate.** The recommended dose of oseltamivir for g**lomerular filtration rate** <30 mL/min/1.73 m^2^ is 2–3 mg/kg/day, based on preliminary data obtained by a National Institutes of Health–funded Collaborative Antiviral Study Group (*10*). The success of our management strategy for this case suggests early treatment with oseltamivir can prevent severe illness in newborns with perinatal influenza A pandemic (H1N1) 2009 infection.

## References

[1] Centers for Disease Control and Prevention. Swine influenza A (H1N1) infection in two children—southern California, March–April 2009. MMWR Morb Mortal Wkly Rep. 2009;58:400–42. 19390508

[2] National Center for Immunization and Respiratory Diseases. CDC; Centers for Disease Control and Prevention. Use of influenza A (H1N1) 2009 monovalent vaccine: recommendations of the Advisory Committee on Immunization Practices (ACIP), 2009. MMWR Recomm Rep. 2009;58(RR-10):1–8. 19713882

[3] Schwartz GJ, Brion LP, Spitzer A. The use of plasma creatinine concentration for estimating glomerular filtration rates in infants, children, and adolescents. Pediatr Clin North Am. 1987;34:571–90. 3588043 10.1016/s0031-3955(16)36251-4

[4] Rowe T, Abernathy RA, Hu-Primmer J, Thompson WW, Lu X, Lim W, Detection of antibody to avian influenza A (H5N1) virus in human serum by using a combination of serologic assays. J Clin Microbiol. 1999;37:937–43. 10074505 10.1128/jcm.37.4.937-943.1999 PMC88628

[5] Irving WL, James DK, Stephenson T, Laing P, Jameson C, Oxford JS, Influenza virus infection in the second and third trimesters of pregnancy: a clinical and seroepidemiological study. BJOG. 2000;107:1282–9. 10.1111/j.1471-0528.2000.tb11621.x 11028582

[6] McGregor JA, Burns JC, Levin MJ, Burlington B, Meiklejohn G. Transplacental passage of influenza A/Bangkok (H3N2) mimicking amniotic fluid infection syndrome. Am J Obstet Gynecol. 1984;149:856–9. 6465250 10.1016/0002-9378(84)90604-5

[7] Yawn DH, Pyeatte JC, Joseph JM, Eichler SL, Garcia-Bunuel R. Transplacental transfer of influenza virus. JAMA. 1971;216:1022–3. 10.1001/jama.216.6.1022 5108246

[8] Purtilo DT, Hallgren HM, Yunis EJ. Depressed maternal lymphocyte response to phytohaemagglutinin in human pregnancy. Lancet. 1972;1:769. 10.1016/S0140-6736(72)90522-3 4111247

[9] Bueva A, Guignard JP. Renal function in preterm infants. Pediatr Res. 1994;36:572–7. 7877873 10.1203/00006450-199411000-00005

[10] Allen U, Blumberg EA, Fischer SA, Green M, Humar A, Ison MG, American Society of Transplantation Infectious Diseases Community of Practice Transplant Infectious Disease Section of the Transplantation Society Guidance on Novel Influenza A/H1N1 [cited 2009 Nov 19]. http://www.transplantation-soc.org/downloads

